# Personalized maps of T1 relaxometry abnormalities provide correlates of disability in multiple sclerosis patients

**DOI:** 10.1016/j.nicl.2023.103349

**Published:** 2023-02-13

**Authors:** Xinjie Chen, Sabine Schädelin, Po-Jui Lu, Mario Ocampo-Pineda, Matthias Weigel, Muhamed Barakovic, Esther Ruberte, Alessandro Cagol, Benedicte Marechal, Tobias Kober, Jens Kuhle, Ludwig Kappos, Lester Melie-Garcia, Cristina Granziera

**Affiliations:** a Translational Imaging in Neurology (ThINK) Basel, Department of Biomedical Engineering, Faculty of Medicine, University Hospital Basel and University of Basel, Basel, Switzerland; b Department of Neurology, University Hospital Basel, Switzerland; c Research Center for Clinical Neuroimmunology and Neuroscience Basel (RC2NB), University Hospital Basel and University of Basel, Basel, Switzerland; d Division of Radiological Physics, Department of Radiology, University Hospital Basel, Basel, Switzerland; e Advanced Clinical Imaging Technology, Siemens Healthineers International AG, Lausanne, Switzerland

**Keywords:** quantitative MRI, Multiple sclerosis, qT1 abnormality maps, EDSS, Expanded Disability Status Scale, NAcGM, normal-appearing cortical grey matter, NAWM, normal-appearing white matter, PPMS, primary progressive multiple sclerosis, RRMS, relapsing-remitting multiple sclerosis, SPMS, secondary progressive multiple sclerosis, WMLs, white matter lesions, GMcLs, cortical grey matter lesions

## Abstract

**Objectives and aims:**

Quantitative MRI (qMRI) has greatly improved the sensitivity and specificity of microstructural brain pathology in multiple sclerosis (MS) when compared to conventional MRI (cMRI). More than cMRI, qMRI also provides means to assess pathology within the normal-appearing and lesion tissue. In this work, we further developed a method providing personalized quantitative T1 (qT1) abnormality maps in individual MS patients by modeling the age dependence of qT1 alterations. In addition, we assessed the relationship between qT1 abnormality maps and patients’ disability, in order to evaluate the potential value of this measurement in clinical practice.

**Methods:**

We included 119 MS patients (64 relapsing-remitting MS (RRMS), 34 secondary progressive MS (SPMS), 21 primary progressive MS (PPMS)), and 98 Healthy Controls (HC). All individuals underwent 3T MRI examinations, including Magnetization Prepared 2 Rapid Acquisition Gradient Echoes (MP2RAGE) for qT1 maps and High-Resolution 3D Fluid Attenuated Inversion Recovery (FLAIR) imaging. To calculate personalized qT1 abnormality maps, we compared qT1 in each brain voxel in MS patients to the average qT1 obtained in the same tissue (grey/white matter) and region of interest (ROI) in healthy controls, hereby providing individual voxel-based Z-score maps. The age dependence of qT1 in HC was modeled using linear polynomial regression. We computed the average qT1 Z-scores in white matter lesions (WMLs), normal-appearing white matter (NAWM), cortical grey matter lesions (GMcLs) and normal-appearing cortical grey matter (NAcGM). Lastly, a multiple linear regression (MLR) model with the backward selection including age, sex, disease duration, phenotype, lesion number, lesion volume and average Z-score (NAWM/NAcGM/WMLs/GMcLs) was used to assess the relationship between qT1 measures and clinical disability (evaluated with EDSS).

**Results:**

The average qT1 Z-score was higher in WMLs than in NAWM. (WMLs: 1.366 ± 0.409, NAWM: −0.133 ± 0.288, [mean ± SD], *p <* 0.001). The average Z-score in NAWM in RRMS patients was significantly lower than in PPMS patients (*p =* 0.010). The MLR model showed a strong association between average qT1 Z-scores in white matter lesions (WMLs) and EDSS (R^2^ = 0.549, β = 0.178, 97.5 % CI = 0.030 to 0.326, *p =* 0.019). Specifically, we measured a 26.9 % increase in EDSS per unit of qT1 Z-score in WMLs in RRMS patients (R^2^ = 0.099, β = 0.269, 97.5 % CI = 0.078 to 0.461, *p =* 0.007).

**Conclusions:**

We showed that personalized qT1 abnormality maps in MS patients provide measures related to clinical disability, supporting the use of those maps in clinical practice.

## Highlights

• The qT1 abnormality map can quantify the single-subject level differences in MS.
• Personalized qT1 abnormalities provide measures related to MS clinical disability.
• qT1 in lesion showed a higher deviation to healthy tissue compared with NA tissue.
• RRMS has milder qT1 abnormalities in NAWM compared with PPMS.

## 1 Introduction

Conventional magnetic resonance imaging (cMRI) provides important individual measures of acute inflammatory activity and global degeneration processes in multiple sclerosis (MS) patients (Fazekas et al., 1999, Gebarski et al., 1985). Nevertheless, cMRI fails to provide MS-related microstructural tissue changes within and outside focal lesions. Those alterations are related to various pathological processes encompassing multifocal inflammatory infiltration, demyelination, microglial activation, degradation of oligodendrocytes and axons, etc. (Reich et al., 2018), which contribute to MS disease progression and disability accrual (Lassmann, 2022).

Quantitative MRI (qMRI) techniques provide measures that are more sensitive and specific to microstructural properties and pathological changes in the central nervous tissue (Granziera et al., 2021, Tofts, 2003). In fact, qMRI allows describing biophysical properties of free/bound water in brain tissue, which may inform about myelin and cellular characteristics (Weiskopf et al., 2021), as well as the concentration of paramagnetic compounds such as iron (Schweser et al., 2011) and the diffusivity of water molecules in different central nervous system (CNS) compartments (Afzali et al., 2021). All these measures may be well altered when MS-typical neuroinflammatory and neurodegenerative processes occur, both in MS lesions and in the “normal-appearing” tissue (Helms, 2015, Granziera et al., 2021).

Yet, establishing the value of qMRI measures in clinical practice remains challenging, owing to the paucity of available quantitative methods in clinical practice, the lack of normative and cut-off values and the scarcity of information related to the association of those measures with clinical tests in single patients (Mohammadi and Callaghan, 2018, Saito et al., 2009).

Compared with group-based analysis, single-subject investigations are more susceptible to statistical and inherent data biases (Hasan et al., 2012a, Hasan et al., 2012b, Martin et al., 2018), which are particularly important in patients affected by neurological pathologies (Arbabshirani et al., 2017). Also, deep-learning-based methods in single subjects are often limited by feature selections, small databases, and overfitting problems (Awan et al., 2021, Seccia et al., 2021). On the other hand, voxel-based quantification (VBQ) (Lommers et al., 2021) approaches offer the opportunity to quantify brain alterations with different qMRI parameters (i.e., MT, R1, and R2*) in individual patients with normalization using the subject-specific deformation field. Nonetheless, voxel-based analysis results are often challenging to interpret, especially in single-to-group studies, because of the limited normalization quality due to rather the large image voxel sizes and the non-isotropic image acquisition, individual differences in neuroanatomy and the inherent risk of false-positive results (Engström et al., 2014, Mechelli et al., 2005, Scarpazza et al., 2013).

Quantitative T1 (qT1) is sensitive to pathological tissue changes in MS patients since alterations in myelin, axons, free water, and iron (e.g., demyelination, axon loss, edema, and chronic inflammation) lead to prolonged T1 relaxation times (Granziera et al., 2021). qT1 mapping also shows high accuracy for the distinction of focal lesions in both white (WM) and grey matter (GM) in MS patients (Kober et al., 2012, Mottershead et al., 2003). At the group level, significant global and focal qT1 changes in MS patients’ brains are also associated with disease progression and the development of brain atrophy (Vrenken et al., 2006a, Vrenken et al., 2006b), with cognitive impairment (Simioni et al., 2014), and with the MS composite scores (Thaler et al., 2015). However, the clinical value of alterations in qT1 maps in single patients has yet to be established.

Previously, we proposed a method to compute individual qMRI deviation maps by comparing one MS patient to a reference distribution of qMRI metrics in healthy tissue (Bonnier et al., 2019). Unlike VBQ and voxel-based morphometry (VBM), this approach allows a voxel-wise comparison in the subject space without inter-patient registration. It can be applied to all qMRI maps, and it is especially accurate when performed on MP2RAGE acquisitions, which provides a time-homogeneous T1 weighting and simultaneous qT1 mapping (Marques et al., 2010). Others previously used qMRI to assess brain tissue abnormalities in single subjects, for example, by (i) using quantitative susceptibility mapping (QSM) deviations in acute brain-injured subjects and healthy controls (Koch et al., 2021), and (ii) evaluating voxel-wise standard Z-score differences in white matter tracts of traumatic brain injury patient compared to healthy subjects (Pannek et al., 2011).

Among the available qMRI methods to assess brain tissue microstructure, qT1 has shown the highest maturity toward integration into the clinical routine (Granziera et al., 2021).

In this study, we further extended the method proposed by Bonnier G. et al. (Bonnier et al., 2019) to quantify single-subject qT1 abnormalities at the voxel level, taking into account the patient's age. Additionally, we explored the clinical relevance of qT1 abnormalities in single subjects by assessing the relationship between changes in normal-appearing (NA) and lesion tissue in patients and patient disability and the group level.

## 2 Methods

### 2.1 Participants

We enrolled 119 MS patients (21 primary progressive MS (PPMS), 64 relapsing-remitting MS (RRMS), 34 secondary progressive MS (SPMS)), and 98 healthy controls (HC) (see Table 1). The inclusion criteria were: (i) MS diagnosis based on the 2017 revisions of McDonald criteria, including RRMS, PPMS, and SPMS subtypes as defined by Lublin et al. (Lublin et al., 2014); (ii) no concurrent Psychiatric or neurological disorder (excluding headache); (iii) absence of contraindication to MRI.

**Table 1:** Characteristics of the study participants.

	MS (n = 119)	MS-GMcLs (n = 85)	RRMS (n = 64)	SPMS (n = 34)	PPMS (n = 21)	HC (n = 98)
Age[mean (SD) , years]	48.3 (13.9)	51.2 (12.9)	39.6 (11.2)	56.7 (9.0)	60.8 (9.5)	36.8 (12.5)
Sex [male/female, n]	47/72	36/49	19/45	15/19	13/8	43/55
Disease duration [mean (SD), months]	11.79 (15.1)	12.16 (12.8)	5.5 (6.5)	20.9 (13.5)	16.3 (25.2)	–
Lesions count [mean (SD), n]	50.8 (39.3)	10.9 (15.1)	45.3 (40.8)	52.4 (24.4)	65.0 (50.5)	–
Lesions volume [median (range),mm^3^]	6795.0 (105.0–66664.0)	134.0 (1956.0- 8.0)	2902.0 (105.0–45410.0)	12224.0 (2186.0–66664.0)	10851.0 (764.0–48258.0)	–
EDSS[median (range) , n]	3.0 (1.5–7.5)	3.5 (1.5–7.5)	2.0 (1.5–7.0)	6.0 (2.0–7.5)	4.0 (2.0–6.5)	–

Data are presented as mean (standard deviation) or median (range) for continuous variables and count for categorical variables. MS: Multiple sclerosis, MS-GMcLs: MS patients with cortical grey matter lesions. RRMS: relapsing-remitting MS, SPMS: secondary progressive MS, PPMS: primary progressive MS, HC: healthy controls, EDSS: Expanded Disability Status Scale, SD: stand deviation. “-” stands for not applicable.

The Ethics Committee northwest/central Switzerland (EKNZ) approved the study, and all participants gave written consent before enrollment.

### 2.2 MRI acquisition

MRI was performed on a 3 T whole-body magnetic resonance system (Magnetom Prisma, Siemens Healthcare, Erlangen, Germany) using the following protocol: (i) High-Resolution 3D Fluid Attenuated Inversion Recovery (FLAIR, TR/TE/TI = 5000/386/1800 ms, voxel size = 1.0 × 1.0 × 1.0 mm^3^, FOV = 256 × 240 × 176 mm^3^, acquisition time = 5:40 min); (ii) Magnetization-Prepared 2 Rapid Acquisition Gradient Echoes (MP2RAGE (Marques et al., 2010), TR/TE/TI1/TI2 = 5000/2.98/700/2500 ms, voxel size = 1.0 × 1.0 × 1.0 mm^3^, FOV = 256 × 240 × 176 mm^3^, acquisition time = 8:20 min). The MP2RAGE protocol provided a T1 weighted image (UNI-MP2RAGE) and T1 relaxometry maps.

### 2.3 Personalized maps of T1 relaxometry abnormalities estimation

**Fig. 1:**
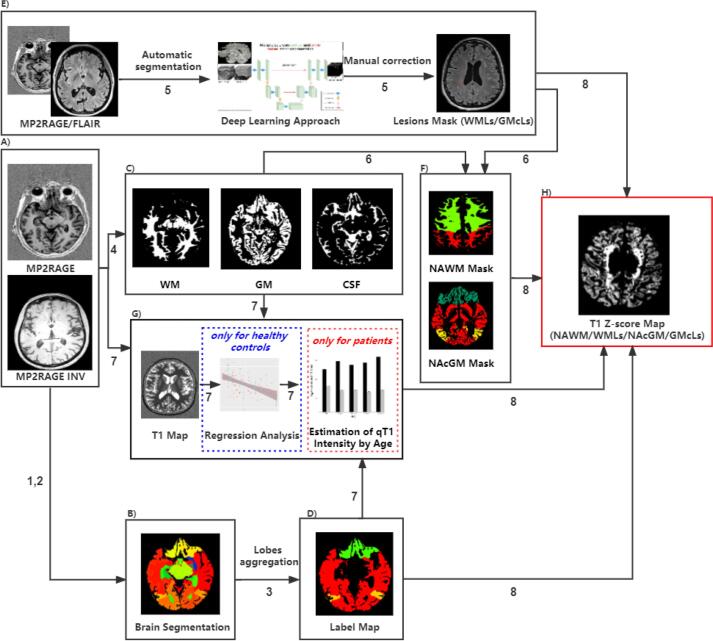
Flowchart of the personalized maps of T1 relaxometry abnormalities computation.

The flowchart of the process to estimate the “ Personalized maps of T1 relaxometry abnormalities” is represented in Fig. 1. It comprised the following steps:

The number along with each arrow corresponds to the order of the process step in personalized maps of T1 relaxometry abnormalities estimation. WM: white matter; GM: grey matter; CSF: cerebrospinal fluid; WMLs: white matter lesions; GMcLs: cortical grey matter lesions; NAWM: normal-appearing white matter; NAcGM: normal-appearing cortex grey matter.

1) Preprocessing: The UNI-MP2RAGE images were skull-stripped in HC and patient groups using the “AI-Rad Companion Brain MR” of Siemens Healthineers (AI-Rad) (Fig. 1, panel A).

2) Brain Segmentation: Brain structure segmentation was performed using the AI-Rad by combining tissue maps with anatomical masks derived from a single-subject template via nonrigid registration (Fig. 1, panel B) (Schmitter et al., 2015). A complete list of brain areas can be found in Supplementary Information, S1.

3) Lobes Aggregation: An in-house python script was implemented to aggregate the brain into five regions of interest (ROIs), including the frontal lobe, parietal lobe, temporal lobe, occipital lobe, and deep grey matter (DGM). The DGM comprises the thalamus, caudate, putamen, and pallidum (Fig. 1, panel D). The frontal lobe, parietal lobe, temporal lobe, and occipital lobe consist of both WM and GM components, while the DGM comprises mainly GM with a small amount of WM.

4) Tissue Concentration Estimation for partial volume effects: A variational expectation–maximization method was used for brain tissue classification in WM, GM, and cerebrospinal fluid (CSF) (Roche et al., 2017) using the UNI-MP2RAGE image. The WM, GM and CSF probability maps were estimated. Partial volume estimation (PVE) was achieved using the approach proposed by Roche and Forbes (2014) (Fig. 1, panel C). The main equation modeling this process was as follows:

(1) $$y=C_{\mathrm{GM}}\omega_{\mathrm{GM}}+C_{\mathrm{WM}}\omega_{\mathrm{WM}}+C_{\mathrm{CSF}}\omega_{\mathrm{CSF}}+\xi ,\;\text{with}\;\xi =N\left(0,\vartheta \right)$$

where, $C_{\mathrm{GM}}$, $C_{\mathrm{WM}}$, $C_{\mathrm{CSF}}$ are GM, WM, and CSF concentrations, respectively; $\omega_{\mathrm{GM}}$, $\omega_{\mathrm{WM}}$, $\omega_{\mathrm{CSF}}$ were the characteristic values of each tissue; ξ is the additive Gaussian noise with zero mean and ϑ standard deviation.

5) Lesion Segmentation and Identification: Automatic segmentation of WM lesions (WMLs) and cortical GM lesions (GMcLs) were performed using a deep-learning 3D U-net method (La Rosa et al., 2020) and further manually corrected by two expert readers. Manual correction of WMLs and GMcLs masks was performed on FLAIR and MP2RAGE, respectively. An in-house python script was used to calculate the number and volume of lesions in the patient cohort, and the results were double-checked by two readers (Fig. 1, panel E).

6) Tissue Mask Computation: WM and GM masks were computed, binarizing the WM and GM probability maps using a threshold of 0.95. We chose the threshold of 0.95 empirically to create accurate tissue masks to the greatest extent, diminishing the partial volume effects. In all cases, it guaranteed to select of those voxels with the highest probability of belonging to CSF, gray, and white matter tissues. The normal-appearing white matter (NAWM) and normal-appearing cortical grey matter (NAcGM) masks were obtained by excluding the voxels belonging to WMLs and GMcLs from the WM and GM masks, respectively (Fig. 1, panel F).

7) qT1 Age-Effect Estimation in Healthy Controls: qT1 biophysical characteristics are relatively homogenous within each cerebral lobe showing a unimodal distribution (Hasan et al., 2012a, Hasan et al., 2012b). Therefore, the mean qT1 value for each ROI (frontal, temporal, parietal, occipital, DGM) in HC was calculated. The qT1 population mean and standard deviation were estimated from the individual mean qT1s previously obtained (Fig. 1, panel G) for GM, WM, and CSF tissues in each ROI represented as: $\mu_{\mathrm{GM}}$, $\mu_{\mathrm{WM}}$, $\mu_{\mathrm{CSF}}$ and $\sigma_{\mathrm{GM}}$, $\sigma_{\mathrm{WM}}$, $\sigma_{\mathrm{CSF}}$. These statistical values derived from each ROI and tissue type were considered reasonably representative of the HC distribution.

The dependence of qT1 with age was modeled using a polynomial linear regression with a maximum order of two as the following equations (Salat et al., 2009, Seiler et al., 2020, Steen et al., 2004):

$$\mu_{\mathrm{GM}}=\alpha_{\mathrm{GM}}*age+\beta_{\mathrm{GM}}*{\mathrm{age}}^{2}+\gamma_{\mathrm{GM}}$$

$$\mu_{\mathrm{WM}}=\alpha_{\mathrm{WM}}*age+\beta_{\mathrm{WM}}*{\mathrm{age}}^{2}+\gamma_{\mathrm{WM}}$$

(2) $$\mu_{\mathrm{CSF}}=\alpha_{\mathrm{CSF}}*age+\beta_{\mathrm{CSF}}*{\mathrm{age}}^{2}+\gamma_{\mathrm{CSF}}$$

where terms $\alpha_{\mathrm{GM}}$, $\alpha_{\mathrm{WM}}$, $\alpha_{\mathrm{CSF}}$ and $\beta_{\mathrm{GM}}$, $\beta_{\mathrm{WM}}$, $\beta_{\mathrm{CSF}}$ were the linear and quadratic coefficients of the polynomial model to be estimated. Terms $\gamma_{\mathrm{GM}}$, $\gamma_{\mathrm{WM}}$, $\gamma_{\mathrm{CSF}}$ were the zero-order coefficients. Term ‘age’ is a vector containing the age of all HC subjects.

The likelihood-ratio test (LRT) was used to choose the most parsimonious model between linear and quadratic polynomials for each ROI.

8) qT1 Deviation Maps Computation: The individualized deviation maps were obtained by calculating the single voxel (*I_v_*) qT1 deviation from the reference qT1 values based on HC using the Z-score measure (Fig. 1, panel H). For each voxel, the mean reference qT1 value and the standard deviation were taken from the corresponding tissue type (WM, GM, or CSF) and the specific ROI the voxel belongs to (frontal, parietal, temporal, and occipital lobes and DGM). The Z-score for a particular voxel ‘v’ was calculated as a variation of the Bonnier et al. (Bonnier et al., 2019) equation as follows:

(3) $$Z_{v}=\frac{C_{\mathrm{GM}}\left(I_{v}-\mu_{\mathrm{GM}}^{\prime }\right)+C_{\mathrm{WM}}\left(I_{v}-\mu_{\mathrm{WM}}^{\prime }\right)+C_{\mathrm{CSF}}\left(I_{v}-\mu_{\mathrm{CSF}}^{\prime }\right)}{\sqrt{\left({C_{\mathrm{GM}}^{2}\sigma }_{\mathrm{GM}}^{2}+C_{\mathrm{WM}}^{2}\sigma_{\mathrm{WM}}^{2}+{C_{\mathrm{CSF}}^{2}\sigma }_{\mathrm{CSF}}^{2}+A+B+C\right)}}$$

where $=2*C_{\mathrm{GM}}C_{\mathrm{WM}}{\mathrm{Cov}}_{\mathrm{GM}/WM}$, $B=2C_{\mathrm{WM}}C_{\mathrm{CSF}}{\mathrm{Cov}}_{\mathrm{WM}/CSF}$, $C=2C_{\mathrm{GM}}C_{\mathrm{CSF}}{\mathrm{Cov}}_{\mathrm{GM}/CSF}$.

$I_{v}$ was the qT1 value of the voxel ‘v’ and $C_{\mathrm{GM}}$, $C_{\mathrm{WM}}$, $C_{\mathrm{CSF}}$ were GM, WM, and CSF tissue concentrations previously calculated in the same voxel. The ${\mathrm{Cov}}_{\mathrm{GM}/WM}$, ${\mathrm{Cov}}_{\mathrm{WM}/CSF}$, and ${\mathrm{Cov}}_{\mathrm{GM}/CSF}$ were the covariances between GM and WM, WM and CSF, GM and CSF in the ROI, the voxel belongs to. Different from Bonnier et al. (Bonnier et al. 2019), we used the reference means $\mu_{\mathrm{GM}}^{\prime }$ , $\mu_{\mathrm{WM}}^{\prime }$ , $\mu_{\mathrm{CSF}}^{\prime }$ obtained from the evaluation of mathematical dependence of qT1 with age in HCs (see Step 7, equation (2)) but using the patient's age ‘age’. Terms $\alpha_{\mathrm{GM}}$, $\alpha_{\mathrm{WM}}$, $\alpha_{\mathrm{CSF}}$ and $\beta_{\mathrm{GM}}$, $\beta_{\mathrm{WM}}$, $\beta_{\mathrm{CSF}}$ are the first and second-order coefficients obtained in equation (2), respectively. Terms $\gamma_{\mathrm{GM}}$, $\gamma_{\mathrm{WM}}$, $\gamma_{\mathrm{CSF}}$ are the zero-order coefficients.

### 2.4 Clinical assessment

MS disease disability was assessed by certified neurologists at Basel University Hospital by using the Neurostatus Expanded Disability Status Scale (EDSS) (https://www.neurostatus.net; Kurtzke, 1983). Patients with EDSS 0 or 1 were excluded from our study due to the log transform applied to EDSS in the statistical analyses. In order to improve the normality before inclusion in the further linear regression analysis, EDSS scores were log-transformed before statistical analysis.

### 2.5 Statistical analysis

In-house scripts to calculate the deviation maps were run under MATLAB© R2021a (https://www.mathworks.com) and Python v3.11 (https://www.python.org). The statistical analysis and visualization were performed using R-project v4.1.1 (https://www.r-project.org).

Inter-group comparisons of the average qT1 Z-scores in NA tissue (NAWM/NAcGM) and lesion tissue (WMLs/GMcLs) were performed among different MS subtypes with ANOVA test followed by Tukey’s multiple comparison test. Intra-group comparisons between the average NAWM Z-score and the average WMLs Z-score or between the average NAcGM Z-score and the average GMcLs Z-score were performed with the paired *t*-test. A two-tailed *p* < 0.05 was considered statistically significant.

Each qT1 deviation map quantifies the parametric variation representative of the changes occurring in an individual brain compared to a distribution of HC, after accounting for patient age using equation (2). We further calculated the mean qT1 Z-scores across all voxels in all patients (i) within the lesions (WMLs/GMcLs) as well as (ii) in NA tissues’ (NAWM/NAcGM). Polynomial linear regression models were used to assess the associations between qT1 and age, followed by the LRT for the model selection. Specifically, the LRT was used for choosing the most parsimonious model between linear and quadratic polynomials for each ROI with a significant level of *p* = 0.05, and null hypothesis H0 of equal data explanation between models. In our case, LRT distributes Chi-square ($\chi_{n}^{2}$) with the degree of freedom *n* = 1.

Linear regression models were used to assess the contribution of (i) average qT1 Z-score within MS lesions (WMLs/GMcLs) and (ii) average qT1 Z-scores within NA tissues (NAWM/NAcGM) to explain the patient disability (EDSS). The multiple linear regression (MLR) model with the backward selection including age, sex, disease duration, phenotype, lesion number, lesion volume, and average Z-score (NAWM/NAcGM/WMLs/GMcLs) as covariates was used to assess the relationship between qT1 abnormalities and EDSS in MS and different MS subtypes.

## 3 Results

### 3.1 Effects of age on qT1

**Fig. 2:**
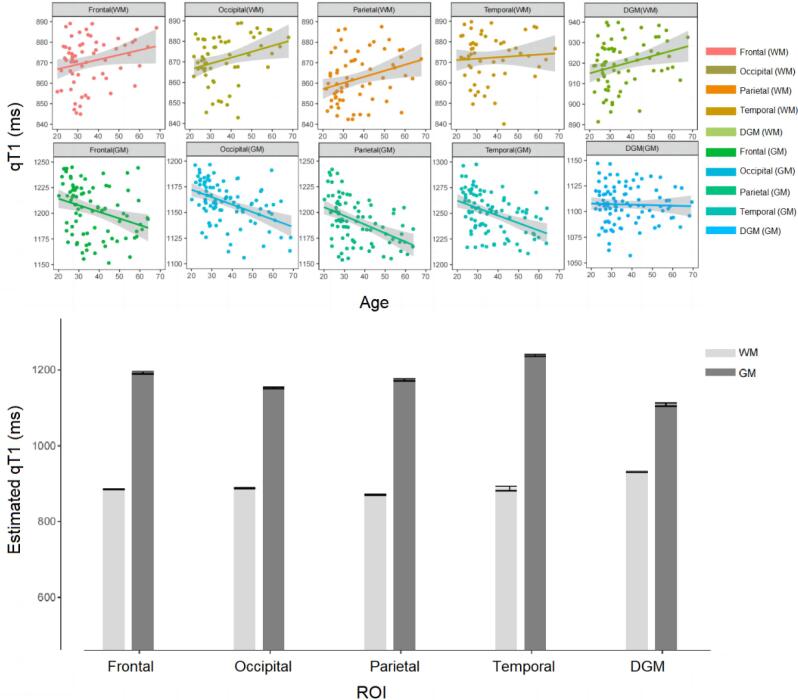
Age regression in healthy controls’ qT1 values.

The qT1 followed a normal distribution in HC in all ROIs. The linear model was more suitable than the quadratic one to explain the dependence of qT1 with age in WM and GM across all ROIs (see Table 2), $\chi^{2}\left(1,N=98\right)<3.842$, *p* > 0.05. The linear polynomial regression models showed statistically significant correlations between age and the average qT1 values in all tissue classes and ROIs, see Table 3 and Fig. 2, panel A.

**Table 2:** The LRT *p*-values for model comparisons.

	Frontal lobe	Parietal lobe	Temporal lobe	Occipital lobe	DGM
*WM*	0.175	0.151	0.138	0.091	0.152
*GM*	0.237	0.183	0.358	0.180	0.251

**Table 3:** Linear regression models of healthy qT1 values and age for all ROIs and tissue classes.

	α	γ	R^2^	*p*
*WM*				
Frontal lobe	0.556	858.603	0.064	0.007
Parietal lobe	0.525	845.268	0.043	0.007
Temporal lobe	–	–	–	–
Occipital lobe	0.529	862.531	0.033	0.040
DGM	0.389	912.446	0.031	0.046
*GM*				
Frontal lobe	−1.211	1250.511	0.202	<0.001
Parietal lobe	−1.268	1234.897	0.251	<0.001
Temporal lobe	−1.109	1291.224	0.275	<0.001
Occipital lobe	−0.934	1197.216	0.229	<0.001
DGM	–	–	–	–

A *p*-value > 0.05 indicates weak evidence against the null hypothesis, suggesting that the linear model was preferred over the quadratic one. DGM (WM) refers to the white matter tissues within the DGM. WM: white matter; GM: grey matter; DGM: deep grey matter.

According to the LRT results, the linear regression model was used to explain the aging effect of qT1 in different tissues across all ROIs. DGM (WM) refers to the white matter tissues within the DGM. WM: white matter; GM: grey matter; DGM: deep grey matter. “-” mean no significant linear dependency.

A) The linear polynomial regression models between age and two types of tissue probability estimation (white matter and grey matter) among different ROIs (frontal lobe, parietal lobe, temporal lobe, occipital lobe, and deep grey matter). B) Bar graphs with error bars (95 % CI) of estimated qT1 values in MS patients. The qT1 values are obtained from the mathematical dependence of reference qT1 values with age in HCs (see Step 7, equation (1)) using the corresponding patient's age. DGM (WM) refers to the white matter tissue dispersing in the WM/GM tissue boundary of the DGM. WM: white matter; GM: grey matter; DGM: deep grey matter; ROI: region of interest.

### 3.2 qT1 abnormalities in MS patients

The average qT1 Z-scores deviation from normality was higher in WMLs than in NAWM (WMLs: 1.366 ± 0.409, NAWM: −0.133 ± 0.288, [mean ± SD], *p <* 0.001), and in GMcLs with respect to NAcGM (GMcLs: 2.089 ± 1.199, NAcGM: 0.304 ± 0.157, [mean ± SD], *p <* 0.001). Furthermore, the average qT1 Z-score in NAWM was significantly lower than in NAcGM (*p <* 0.001), and the average qT1 Z-score in WMLs was significantly lower than in GMcLs (*p <* 0.001) (see Table 4).

**Table 4:** Average Z-scores distribution in MS cohort and subgroup.

	MS	RRMS	SPMS	PPMS
n	119	64	34	21
Average Z-scores in NAWM [mean (SD)]	−0.133 (0.288)	−0.219 (0.252)	−0.044 (0.293)	−0.133 (0.311)
Average Z-scores in WMLs [mean (SD)]	1.366 (0.409)	1.359 (0.464)	1.416 (0.367)	1.308 (0.277)
MS-GMcLs (n)	85	43	28	14
Average Z-scores in NAcGM [mean (SD)]	0.304 (0.157)	0.249 (0.115)	0.345 (0.180)	0.392 (0.169)
Average Z-scores in GMcLs [mean (SD)]	2.089 (1.199)	2.387 (1.190)	1.749 (1.310)	1.852 (0.745)

MS: Multiple sclerosis; MS-GMcLs: Multiple sclerosis patients with cortical grey matter lesions; RRMS: relapsing-remitting MS; SPMS: secondary progressive MS; PPMS: primary progressive MS; NAWM: normal-appearing white matter; NAcGM: normal-appearing cortical grey matter.

**Fig. 3:**
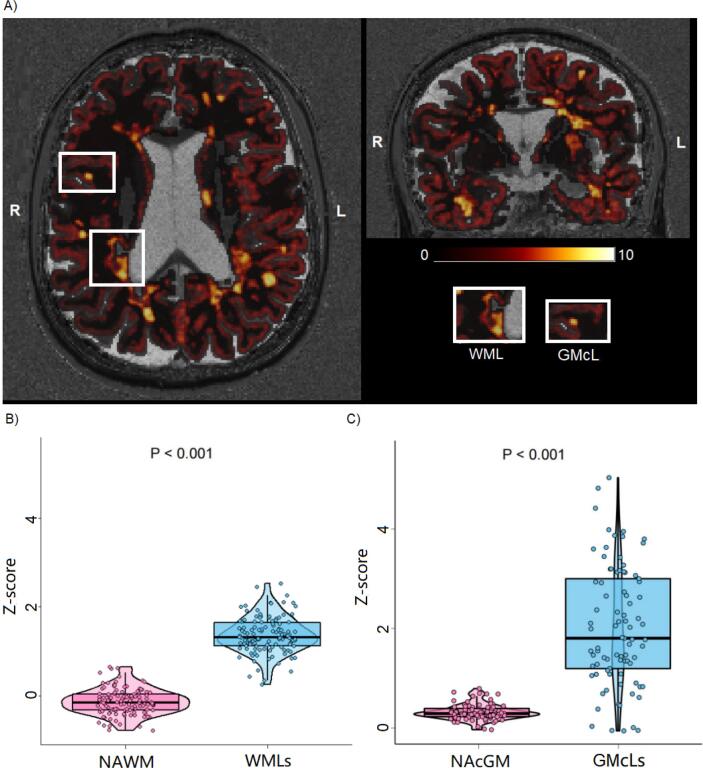
Qt1 abnormality maps (z-scores qt1 maps).

The average Z-score in NAWM in RRMS patients was significantly lower than in PPMS patients (*p =* 0.010) and SPMS patients (*p =* 0.009), while the average NAWM Z-scores in PPMS and SPMS were not significantly different. No statistical difference was observed in the subgroup comparisons for the average Z-scores in WMLs. Further group comparisons only showed a significant decrease in the mean Z-score in NAcGM in the RRMS group compared with the PPMS group (*p =* 0.007) and the SPMS group (*p =* 0.025) (see Table 4). The contrast of average qT1 Z-score maps in WMLs and NAMW can be visualized qualitatively in Fig. 3.

A) Axial/coronal view of a qT1 Z-score map. Zoomed area showed the views of qT1 Z-score in one representative WML and CL. The color bar represented the range of Z-score values (from 0.01 to 10.00 in this qT1 Z-score map). B) Average qT1 Z-scores group comparison between WMLs and NAWM. C) Average qT1 Z-scores group comparison between NAcGM and GMcLs. L: left; R: right; NAWM: normal-appearing white matter; NAcGM: normal-appearing cortex; WMLs: white matter lesions; GMcLs: cortical grey matter lesions.

### 3.3 Correlation between qT1 abnormalities and disability in MS patients

**Fig. 4:**
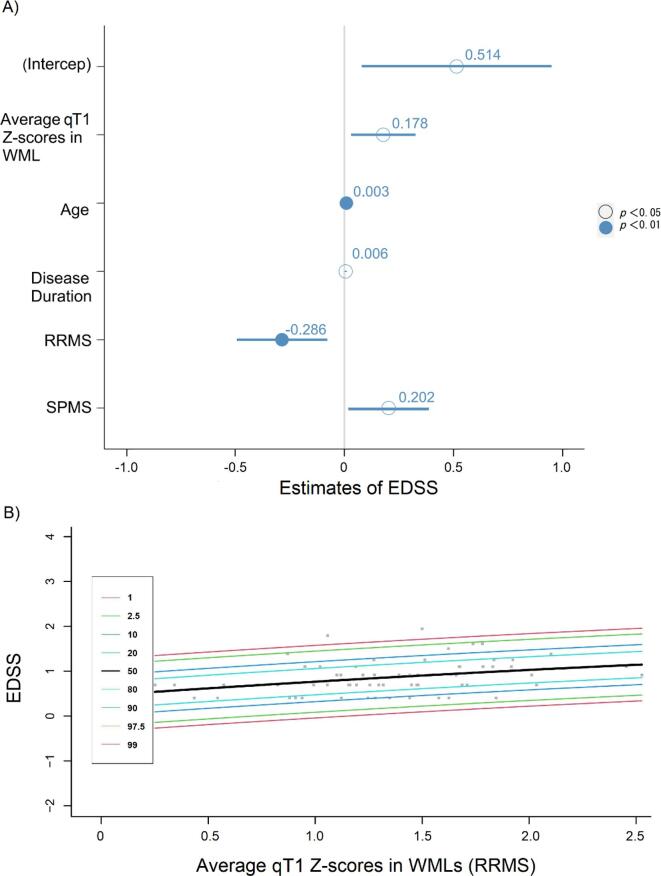
Relationship between Z-scores in WMLs and EDSS.

A multiple linear regression model with backward selection showed that qT1 Z-scores in WMLs (but not in NAWM) were significantly related to clinical disability in MS patients (R^2^ = 0.549, F (5, 113) = 29.77, β = 0.514, *p* = 0.021, see Table 5). The integrated model equation was: EDSS = 0.514 + 0.009 * Age + 0.006 * Disease Duration + 0.178 * average qT1 Z-score in WMLs − 0.286* Diagnosis (RRMS) + 0.202* Diagnosis (SPMS) + 0.514. The forest plot of results is shown in Fig. 4, panel A.

**Table 5:** Multiple linear regression analysis in MS cohort and subgroup.

	β	Lower 95 %	Upper 95 %	SE	*t* Stat	*p*
MS (n = 119)						
(Intercept)	0.514	0.079	0.949	0.219	2.343	0.021
age	0.009	0.003	0.015	0.003	3.034	0.003
Disease duration	0.006	0.001	0.010	0.002	2.468	0.015
Average qT1 Z-score (WMLs)	0.178	0.030	0.326	0.075	2.389	0.019
Diagnosis						
*RRMS*	−0.286	−0.493	−0.079	0.105	2.735	0.007
*SPMS*	0.202	0.018	0.387	0.093	2.178	0.032
*PPMS (reference)*	–	–	–	–	–	–
RRMS (n = 72)						
(Intercept)	0.494	0.219	0.769	0.138	3.594	0.001
Average qT1 Z-score (WMLs)	0.269	0.078	0.461	0.096	2.809	0.007

Diagnosis as PPMS was selected as the reference category variable to create dummy variables in the multiple linear regression model in the MS cohort. MS: multiple sclerosis; RRMS: relapsing-remitting multiple sclerosis; SPMS: secondary progressive MS; PPMS: primary progressive MS; SE: standard error. “-” mean no significant linear dependency.

A) A forest plot of multiple linear regression analysis results: age (β = 0.003, *p* = 0.003), disease duration (β = 0.006, *p* = 0.015), average qT1 z-scores in WMLs (β = 0.178, *p* = 0.019), diagnosis (RRMS) (β = -0.286, *p* = 0.007), and diagnosis (SPMS) (β = 0.202, *p* = 0.032) were independence variables, EDSS was dependence variable. B) Centile curves for separate ranges of the EDSS scores based on the estimated average qT1 Z-score in WMLs for distributions belonging to the subgroup GLM model (RRMS). The fitted linear regression model was: EDSS = 0.269 * average qT1 Z-score in WMLs + 0.494. Colored lines represent different centile levels. RRMS: relapsing-remitting multiple sclerosis, EDSS: Expanded Disability Status Scale.

In RRMS patients, we found a statistically significant linear relationship (R^2^ = 0.099, F (1, 62) = 7.88, p < 0.01) between the average qT1 Z-score in WML and EDSS (see Table 5). The centile curves of the results are shown in Fig. 4, panel B. No statistical significance in linear regression analysis between average Z-score maps and EDSS was observed in both the PPMS and the SPMS groups.

In MS patients with GMcLs, no significant statistical correlation was found between the average qT1 Z-score in NAcGM/GMcLs and EDSS.

## 4 Discussion

In this work, we assessed the clinical value of personalized maps of qT1 abnormalities by investigating their relationship with clinical disability scores (evaluated with EDSS) in MS patients. To achieve this goal, we further developed a method we had previously proposed to calculate qT1 abnormalities in single patients (Bonnier et al., 2019) by (i) increasing the number of healthy controls to achieve more generalizable results and increased statistical power; (ii) assessing deviation maps also in the cortex and cortical lesions; and estimating the effect of aging on the distribution of qT1 values in healthy controls. Our results showed a significant association between average qT1 Z-scores in WMLs in MS patients and EDSS, suggesting that the single patient's qT1 abnormality maps might be used in clinical practice to monitor disease evolution.

Different than qMRI measures, quantitative abnormality maps permit quantifying to which extent a qMRI measure is abnormal compared to a large group of healthy subjects (Bonnier et al., 2019). In addition, they allow the precise quantification of the localization and extent of damage, which clinical tests do not provide.

Applying this modified approach to obtain personalized maps of T1 relaxometry abnormalities, we showed that MS patients exhibit a higher average qT1 Z-score in lesions (WMLs/GMcLs) compared to the average Z-score in NA tissue (NAWM/NAcGM). These results confirm preliminary evidence (Bonnier et al., 2014, Bonnier et al., 2019) and point to a loss of brain tissue integrity (i.e., axonal, myelin, and cell damage) and/or extracellular water accumulation (Brück et al., 2002, Lassmann et al., 2001, Metz et al., 2014) in lesions compared to non-lesion tissue.

Interestingly, while the average qT1 Z-score in WMLs and GMcLs were similar between RRMS and progressive patients, the average NAWM Z-score and the average NAcGM Z-score were higher in progressive than in RRMS subjects. These findings confirm previous work showing that surrogate measures of myelin and axon integrity were reduced in the progressive vs RRMS population using qMRI metrics including neurite density index (NDI) and myelin water fraction (MWF) (Rahmanzadeh et al., 2021). These results were also coherent with previous pathological studies (Lassmann, 2022), which showed similar lesion damage between RRMS and progressive MS patients but increased normal-appearing tissue damage in the progressive forms. In RRMS patients, the milder alteration in qT1 measured in NAWM compared to progressive patients might result from less pronounced tissue degeneration and reparative processes like remyelination and gliosis (Granziera et al., 2021, Rahmanzadeh et al., 2021). On the other hand, the mild increase in qT1 in NAcGM might be the result of iron deposition in the cortex (Parry et al., 2003), which was also reported by previous studies in RRMS patients, but not in SPMS or PPMS (Griffin et al., 2002a, Griffin et al., 2002b).

Multiple linear regression analysis showed a significant correlation between qT1 Z-scores in WMLs, together with age, disease duration, and disease subtype. Interestingly, there was a strong relationship between qT1 Z-scores and EDSS in RRMS patients. To our surprise, however, the mild diffuse neurodegeneration measured in NAWM and NAcGM was unrelated to patients' EDSS. The lack of association might depend on the fact that we averaged qT1 z-scores across the entire NAWM (Bonnier et al., 2019) and/or on the milder nature of the damage in the white matter/grey matter tissue outside the lesions (Inglese and Bester, 2010). On the other hand, the coarse nature of the EDSS might also have contributed to the absence of correlation (Barnett et al., 2020). Further studies should explore the regional impact of those abnormalities (i.e., whether they have a more significant effect on patient disability if they occur in brain regions where major fiber bundles are found, around the ventricles vs the juxtacortical areas, etc.).

In clinical practice, we lack methods that provide personalized evaluations of focal and diffuse tissue damage severity. The current approach might overcome this challenge since it allows achieving measures that strongly relate to patient disability. Compared to “atlas”-based methods (Dvorak et al., 2021, Piredda et al., 2020, Shah et al., 2022), the current one has the advantage of minimizing partial volume effects and allowing a more accurate and sensitive estimation of age-independent damage. Of course, the path toward a possible clinical integration is still long (Granziera et al., 2021, Press et al., 2018), as it will require (i) further expansion of the current healthy control population for increased generalizability; (ii) the assessment of reproducibility across MRI sites and (iii) the integration of the methodology in the clinical workflow. Besides, the current approach may be expanded to other qMRI contrasts, increasing the specificity for damage to specific CNS components, such as axons, myelin, or cells (Gouw et al., 2011, Weiskopf et al., 2021). Furthermore, the presented work could be easily extended to other neurological disorders like cognitive impairment, neuroHIV infection, and migraine since the applied qMRI metrics are sensitive to brain tissue alterations related to neuroinflammation and neurodegeneration pathological changes (Granziera et al., 2014, Granziera et al., 2015, Granziera et al., 2021).

However, we did not find any correlation between average qT1 abnormalities and EDSS in both PPMS and SPMS patients. This might well be due to the low sample size of these two groups, which indicated studies should be adequately powered for these MS subtypes in order to confirm or not these findings. Future scan-rescan and longitudinal studies should also assess individual variability and single-subject predictions of the presented methodology. Additional work should also explore the sensitivity of personalized maps of T1 relaxometry abnormalities for monitoring disease progression and individual sensitivity to treatment in areas of focal inflammation/degeneration and diffuse damage.

In summary, our study provided evidence that personalized qT1 abnormalities maps relate to clinical disability in MS patients. These results open the path toward the application of this methodology in the clinical management of MS patients. Future work will expand current findings toward clinical integration, multicentric assessment, single-subject predictions and longitudinal evaluation of the current approach.

## CRediT authorship contribution statement

**Xinjie Chen:** Conceptualization, Methodology, Software, Data curation, Visualization, Writing – original draft, Writing – review & editing. **Sabine Schädelin:** Methodology, Data curation, Conceptualization. **Po-Jui Lu:** Methodology, Formal analysis. **Mario Ocampo-Pineda:** Methodology, Data curation. **Matthias Weigel:** Conceptualization, Formal analysis. **Muhamed Barakovic:** Methodology, Formal analysis. **Esther Ruberte:** Methodology, Validation. **Alessandro Cagol:** Methodology, Validation. **Benedicte Marechal:** Software. **Tobias Kober:** Software. **Jens Kuhle:** Writing – review & editing. **Ludwig Kappos:** Writing – review & editing. **Lester Melie-Garcia:** Supervision, Writing – review & editing, Conceptualization, Formal analysis, Methodology. **Cristina Granziera:** Supervision, Project administration, Funding acquisition, Writing – review & editing, Conceptualization, Methodology.

## Declaration of Competing Interest

The authors declare that they have no known competing financial interests or personal relationships that could have appeared to influence the work reported in this paper.

## Data Availability

The data that has been used is confidential.
