# Supplementary material for: Personalized maps of T1 relaxometry abnormalities provide correlates of disability in multiple sclerosis patients
Source: Neuroimage Clin. 2023 Feb 13;37:103349. doi: 10.1016/j.nicl.2023.103349 (PMC9958406; doi:10.1016/j.nicl.2023.103349)
Supplement: Supplementary Data 1 [file mmc1.docx]

**Supplementary material**

S1 Full List of Segmentation Labels

| Index | Label Description |
| --- | --- |
| 1 | CSF |
| 2 | GM |
| 3 | WM |
| 4 | White abnormal |
| 5 | Thalamus (L) |
| 6 | Thalamus (R) |
| 7 | Caudate (L) |
| 8 | Caudate (R) |
| 9 | Putamen (L) |
| 10 | Putamen (R) |
| 11 | Pallidum (L) |
| 12 | Pallidum (R) |
| 13 | Hippocampus (L) |
| 14 | Hippocampus (R) |
| 15 | Lateral ventricle (L) |
| 16 | Lateral ventricle (R) |
| 17 | Third ventricle |
| 18 | Fourth ventricle |
| 19 | Pons |
| 20 | Mesencephalon |
| 21 | Medulla oblongata |
| 22 | Frontal (L) (GM) |
| 23 | Frontal (L) (WM) |
| 24 | Frontal (R) (GM) |
| 25 | Frontal (R) (WM) |
| 26 | Parietal (L) (GM) |
| 27 | Parietal (L) (WM) |
| 28 | Parietal (R) (GM) |
| 29 | Parietal (R) (WM) |
| 30 | Occipital (L) (GM) |
| 31 | Occipital (L) (WM) |
| 32 | Occipital (R) (GM) |
| 33 | Occipital (R) (WM) |
| 34 | Temporal (L) (GM) |
| 35 | Temporal (L) (WM) |
| 36 | Temporal (R) (GM) |
| 37 | Temporal (R) (WM) |
| 38 | Cingulate (L) (GM) |
| 39 | Cingulate (R) (GM) |
| 40 | Corpus callosum (L) (WM) |
| 41 | Corpus callosum (R) (WM) |
| 42 | Insula (L) |
| 43 | Insula (R) |
| 44 | Deep white matter (L) |
| 45 | Deep white matter (R) |
| 46 | Cerebellum (L) (GM) |
| 47 | Cerebellum (L) (WM) |
| 48 | Cerebellum (R) (GM) |
| 49 | Cerebellum (R) (WM) |
| 50 | Amygdala (L) |
| 51 | Amygdala (R) |

WM: white matter, GM: grey matter, CSF: cerebrospinal fluid, L: left, R: right.
